# Bioactive 3-Decalinoyltetramic Acids Derivatives From a Marine-Derived Strain of the Fungus *Fusarium equiseti* D39

**DOI:** 10.3389/fmicb.2019.01285

**Published:** 2019-06-07

**Authors:** Donglin Zhao, Xiaobin Han, Dan Wang, Minghong Liu, Jianyu Gou, Yulong Peng, Jing Liu, Yiqiang Li, Fei Cao, Chengsheng Zhang

**Affiliations:** ^1^Marine Agriculture Research Center, Tobacco Research Institute of Chinese Academy of Agricultural Sciences, Qingdao, China; ^2^Zunyi Branch, Guizhou Tobacco Company, Zunyi, China; ^3^College of Pharmaceutical Sciences, Hebei University, Baoding, China

**Keywords:** 3-decalinoyltetramic acid, fusarisetin, phytotoxicity, anti-phytopathogenic activity, OSMAC fermentation

## Abstract

Two novel 3-decalinoyltetramic acid (3DTA) derivatives, namely fusarisetins C and D (**1** and **2**), and four known derivatives (**3–6**) were isolated from the marine-derived fungus *Fusarium equiseti* D39. Their structures were determined by spectroscopic data, vibrational circular dichroism (VCD) calculations, and X-ray crystallography. Compound **2** was identified as the first fusarisetin to possess an unprecedented carbon skeleton with a tetracyclic ring system comprised of a decalin moiety (6/6) and a tetramic acid moiety. A plausible biosynthetic pathway for the isolated compounds was proposed. All 3DTAs derivatives exhibited a potent phytotoxicity, and **5** also displayed a remarkable anti-phytopathogenic activity superior to the positive control resulting in damage of the cell membrane of *Pseudomonas syringae* and ensuing leakage of the intracellular components. Here, the phytotoxicity of fusarisetins has been reported for the first time. The OSMAC fermentation optimization approach to give **5** was performed by varying the culture media and salinities. The results showed that potato liquid medium with 1% salinity is the most favorable condition for the production of **5**.

## Introduction

3-Decalinoyltetramic acids (3DTAs) are natural products that contain the tetramic acid structure (pyrrolidine-2,4-dione) featuring substituted decalin ring systems (Schobert and Schlenk, 2008; Mo et al., 2014). The most representative compound is equisetin, which was the first member of this family to be identified (Royles, 1995). 3-Decalinoyltetramic acids have been isolated from various terrestrial and marine microorganisms, but mainly from fungi (Mo et al., 2014). These compounds exhibited various notable biological activities, such as antimicrobial, antiviral, cytotoxic, and phytotoxic activities (Schobert and Schlenk, 2008; Mo et al., 2014). In 2011, the biogenetically related compound fusarisetin A, that possesses both an unprecedented carbon skeleton and a new pentacyclic ring system with acinar morphogenesis inhibitory activity, was identified, thereby enriching the structural and biological diversity of 3DTAs (Jang et al., 2012). Thus, due to their intriguing structures and associated biological activities, 3DTAs are gaining growing attention from the communities of biologists and chemists.

Marine fungi are a hotspot for the study of various natural products, as they produce secondary metabolites with novel structures and interesting bioactivities endowed by the unique marine environment (Carroll et al., 2019). However, each microbial strain has the potential to produce multiple compounds, and only subsets of these compounds are produced under specific growth conditions. Based on this, the “one strain many compounds” (OSMAC) culture strategy was conceptualized to increase the chemical diversity and improve the yields of new microbial bioactive compounds by varying the nutrient content, temperature, salinity, aeration, etc. (Romano et al., 2018).

**FIGURE 1:**
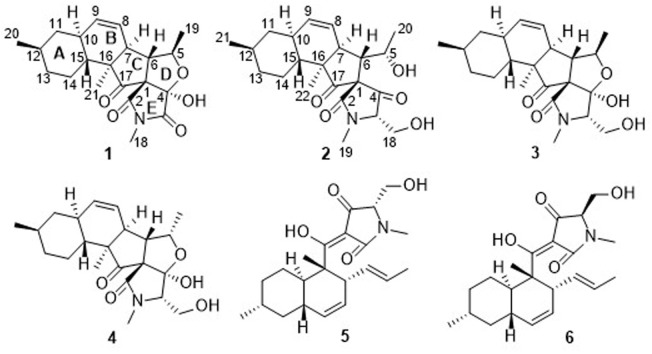
Chemical structures of **1–6**.

During our ongoing search for novel secondary metabolites with agricultural bioactivities from marine-derived fungi (Huang et al., 2018; Zhao et al., 2018), the fungus *Fusarium equiseti* D39 (previously numbered as GLY27 and P18), collected from the intertidal zones of the Yellow Sea in Qingdao, China, attracted our attention because the extract of the fungal culture exhibited a strong anti-phytopathogenic activity. Using the bioassay-LCMS-^1^H NMR screening technology, the HPLC profile and ^1^H NMR spectrum of the extract of the fungal culture were obtained and found to exhibit distinctive UV-absorption peaks and proton signals corresponding to 3DTAs, while the MS spectrum indicated the presence of some novel 3DTAs. However, only two anthraquinone derivatives were isolated from the fungal cultures (Zhao et al., 2018), thereby prompting further investigations into the metabolome of this fungus to isolate the 3DTAs. Further chemical investigation of the ethyl acetate (EtOAc) extracts led to the isolation of six 3DTAs (Figure 1), including two novel fusarisetins, namely fusarisetins C and D (**1** and **2**), and the four known compounds fusarisetin B (**3**), fusarisetin A (**4**), equisetin (**5**), and epi-equisetin (**6**). To the best of our knowledge, only two fusarisetins have been reported as natural products to date (Ahn et al., 2012; Jang et al., 2012). Thus, we herein report the isolation, structural elucidation, and biological activities of these compounds. In addition, to improve the yield of compound **5**, fermentation optimization was carried out using the OSMAC approach.

## Materials and Methods

### General Experimental Procedures

Optical rotations were measured on a JASCO P-1020 digital polarimeter with a 1 dm cell (Jasco, Inc., Easton, MD, United States). UV spectra were recorded on a Techcomp UV2310II spectrophotometer (Techcomp, Ltd., Shanghai, China). IR and vibrational circular dichroism (VCD) spectra were acquired using a BioTools ChiralIR-2X spectrophotometer (BioTools Inc., Olathe, KS, United States). NMR spectra were acquired on an Agilent DD2 500 MHz NMR spectrometer (500 MHz for ^1^H and 125 MHz for ^13^C; Agilent Technologies, Santa Clara, CA, United States), using tetramethylsilane (TMS) as an internal standard. Electrospray ionization mass spectrometry (ESIMS) and high resolution ESIMS (HRESIMS) were carried out using a Micromass Q-TOF spectrometer (Waters, Milford, MA, United States) and a Thermo Scientific LTQ Orbitrap XL spectrometer (Thermo Fisher Scientific, Waltham, MA, United States). Single-crystal data were collected on an Aglient Technologies Gemini E Ultra system (Cu Kα radiation) (Agilent Technologies). Semi-preparative HPLC was performed on a C_18_ (Waters, 5 μm, 10 × 250 mm) column using a Waters e2695 separation module equipped with a Waters 2998 detector (Waters). Silica gel (200–300 mesh; Qing Dao Hai Yang Chemical Group Co., Qingdao, China), octadecylsilyl silica gel (ODS) (RP18, 40–63 μm; Merck, Billerica, MA, United States), and Sephadex LH-20 (GE Healthcare, Pittsburgh, PA, United States) were used for column chromatography. Compounds were monitored by thin layer chromatography (TLC) (G60, F-254; Yan Tai Zi Fu Chemical Group Co., Yantai, China), and spots were visualized by heating the silica gel plates sprayed with 12% H_2_SO_4_ in H_2_O containing saturated vanillins. All the solvents for extraction and isolation were of analytical and HPLC grade.

### Fungal Material

The fungal strain *F. equiseti* D39 was isolated from a piece of fresh tissue obtained from the inner part of an unidentified plant, which was collected from the intertidal zone of the Yellow Sea, Qingdao, China, in July 2016. The fungus was identified according to its morphological characteristics and a molecular protocol by amplification and sequencing of the DNA sequences of the ITS region of the rDNA gene (Zhao et al., 2018). The strain was deposited in the Marine Agriculture Research Center, Tobacco Research Institute of Chinese Academy of Agricultural Sciences, Qingdao, China, with the GenBank (NCBI) accession number KY945342.

### Extraction and Isolation

The fungal strain *F. equiseti* D39 was fermented by solid-state fermentation (SSF) on rice medium in 100 Erlenmeyer flasks (each containing 80 g of rice and 120 mL of H_2_O) at 28° C for 30 days. The culture medium was extracted three times repeatedly with EtOAc, and the solvent was concentrated under reduced pressure to yield the EtOAc extract (25.8 g). This EtOAc extract was then subjected to vacuum liquid chromatography (VLC) on silica gel using a step gradient elution of EtOAc–petroleum ether (0–100%) and subsequently EtOAc–MeOH (0–100%) to provide six fractions (Fr.1–Fr.6). Fraction 3 was initially fractionated using an ODS gel column with a step gradient elution of MeOH–H_2_O (30–90%), followed by separation on Sephadex LH-20 CC (CH_2_Cl_2_/MeOH, v/v, 1/1) to afford Fr. 3-1–Fr. 3-8. Fraction 3-6 was then purified by reversed phase (RP)-HPLC eluting with 55% MeOH–H_2_O to obtain compound **1** (5.0 mg). Fraction 4 was separated on silica gel CC (EtOAc/petroleum ether = 20/80 to 100/0) to obtain Fr. 4-1–Fr. 4-5. Fraction 4-2 was applied to an ODS column eluting with 30–90% MeOH–H_2_O, followed by purification on Sephadex LH-20 CC (CH_2_Cl_2_/MeOH, v/v, 1/1) to obtain Fr. 4-2-1–Fr. 4-2-9. Fraction 4-2-6 was further separated by HPLC using 40% MeCN–(H_2_O + 0.1% TFA) to yield compounds **3** (61.2 mg) and **4** (28.5 mg). Fraction 4-2-7 was purified by HPLC using 55% MeCN–H_2_O (containing 0.1% TFA) to give compounds **5** (238.2 mg) and **6** (126.5 mg). Fraction 5 was subjected to ODS column chromatography using a gradient elution of 30–80% MeOH–H_2_O to afford subfractions Fr.5-1–Fr.5-8. Fraction 5-7 was then subjected to chromatography on Sephadex LH-20 (CH_2_Cl_2_–MeOH, v/v, 1/1) and finally purified by semipreparative HPLC eluting with 60% MeOH–H_2_O to give compound **2** (4.0 mg).

Fusarisetin C (**1**): Colorless crystals; [α]^20^_D_ +15.0 (*c* 0.25, MeOH); UV (MeOH) λ_max_ (log ε) 202 (3.42) nm; ^1^H and ^13^C NMR data, Tables 1, 2; HRESIMS *m/z* 372.1825 [M - H]^−^ (calcd for C_21_H_27_NO_5_, 372.1816).

**Table 1:** ^1^H NMR Data (500 MHz, δ in ppm, *J* in Hz) and ^13^C NMR Data (125 MHz, δ in ppm) for **1**.

	1 (CD_3_OD)		1 (DMSO-*d*_6_)	
Position	δ_C_, type	δ_H_ (*J* in Hz)	δ_C_, type	δ_H_ (*J* in Hz)
1	75.3		73.3	
2	174.0		172.6	
3	174.6		172.9	
4	106.9		105.5	
5	85.4	4.74 (dq, *J* = 6.5, 2.0 Hz)	82.8	4.74 (dq, *J* = 6.5, 1.5 Hz)
6	58.8	2.73 (dd, *J* = 10.5, 2.0 Hz)	56.8	2.63 (dd, *J* = 10.5, 1.5 Hz)
7	50.6	2.48 (dd, *J* = 10.5, 4.5 Hz)	48.3	2.42 (dd, *J* = 10.5, 4.5 Hz)
8	125.3	5.84 (ddd, *J* = 10.0, 4.5, 2.5 Hz)	124.5	5.86 (ddd, *J* = 10.0, 4.5, 2.0 Hz)
9	133.5	5.59 (d, *J* = 10.0 Hz)	131.8	5.54 (d, *J* = 10.0 Hz)
10	37.9	1.91-1.93 (m)	35.9	1.86-1.88 (m)
11	43.1	1.88-1.90 (m)	41.3	1.84-1.86 (m)
		0.84 (q, *J* = 12.5 Hz)		0.74–0.78 (m)
12	34.1	1.44-1.53 (m)	32.2	1.46 (m)
13	36.4	1.76 (m)	34.8	1.71 (brd, *J* = 12.0 Hz)
		0.91-0.98 (m)		0.81-0.85 (m)
14	26.3	1.44-1.53 (m)	24.7	1.36-1.39 (m)
		1.09-1.16 (m)		1.01-1.09 (m)
15	39.1	1.44-1.53 (m)	37.4	1.32-1.34 (m)
16	55.2		53.2	
17	210.8		209.0	
18	25.4	3.02 (s)	25.0	2.96 (s)
19	23.2	1.15 (d, *J* = 6.5 Hz)	22.6	1.06 (d, *J* = 6.5 Hz)
20	22.7	0.94 (d, *J* = 6.5 Hz)	22.2	0.88 (d, *J* = 5.5 Hz)
21	14.3	0.98 (s)	13.8	0.88 (s)
4-OH				8.17 (s)

**Table 2:** ^1^H NMR Data (500 MHz, δ in ppm, *J* in Hz) and ^13^C NMR Data (125 MHz, δ in ppm) for **2**.

	2 (CD_3_OD)		2 (DMSO-*d*_6_)	
Position	δ_C_, type	δ_H_ (*J* in Hz)	δ_C_, type	δ_H_ (*J* in Hz)
1	73.9		71.7	
2	171.7		168.3	
3	71.2	4.12 (dd, *J* = 5.0, 3.0 Hz)	69.7	4.06 (dd, *J* = 6.0, 3.0 Hz)
4	204.2		203.8	
5	68.1	4.08 (m)	65.8	3.94 (m)
6	58.5	2.64 (brd, *J* = 11.5 Hz)	57.3	2.45 (dd, *J* = 11.5, 9.5 Hz)
7	47.1	2.67 (brd, *J* = 11.5 Hz)	45.2	2.59 (dd, *J* = 11.5, 5.0 Hz)
8	126.4	5.87 (m)	125.5	5.82 (m)
9	133.6	5.58 (d, *J* = 10.0 Hz)	131.8	5.53 (brd, *J* = 10.0 Hz)
10	38.2	1.86 (brd, *J* = 12.0 Hz)	36.2	1.80 (brd, *J* = 11.0 Hz)
11	42.9	1.89 (brd, *J* = 12.0 Hz)	41.2	1.85 (brd, *J* = 11.0 Hz)
		0.83 (m)		0.74 (m)
12	34.1	1.48 (m)	32.1	1.46 (m)
13	36.4	1.74 (m)	34.8	1.72 (brd, *J* = 12.5 Hz)
		0.90 (m)		0.79 (m)
14	26.5	1.32 (m)	24.8	1.24 (m)
		1.11 (m)		1.03 (m)
15	39.0	1.52 (dt, *J* = 10.5, 2.0 Hz)	37.2	1.39 (dt, *J* = 12.0, 2.0 Hz)
16	55.6		53.5	
17	210.6		210.1	
18	61.5	3.93 (dd, *J* = 12.0, 3.5 Hz)	61.3	3.75 (m)
		3.83 (dd, *J* = 12.0, 4.5 Hz)		3.61 (m)
19	28.6	3.11 (s)	23.8	1.15 (d, *J* = 6.5 Hz)
20	24.1	1.26 (d, *J* = 6.5 Hz)	28.1	2.99 (s)
21	22.7	0.93 (d, *J* = 6.5 Hz)	22.2	0.87 (d, *J* = 9.0 Hz)
22	15.4	0.98 (s)	14.8	0.86 (s)
5-OH				4.98 (d, *J* = 5.0 Hz)
18-OH				4.94 (m)

Fusarisetin D (**2**): Colorless oil; [α]^20^_D_ -9.2 (*c* 0.13, MeOH); UV (MeOH) λ_max_ (log ε) 200 (3.19) nm; ^1^H and ^13^C NMR data, Tables 1, 2; HRESIMS *m/z* 390.2278 [M + H]^+^ (calcd for C_22_H_32_NO_5_, 390.2275).

X-ray crystallographic analysis of **1** and **3**. Colorless crystals of **1** and **3** were obtained upon evaporation of the CH_2_Cl_2_–MeOH (2:1, v/v) solvent mixture by maintaining the sample at 25°C for 3 days. Single-crystal X-ray diffraction data were recorded on a Xcalibur, Eos, Gemini ultra diffractometer at 298 K. The structures were solved by direct methods (SHELXS-2018) and refined using full-matrix least-squares difference Fourier techniques. All non-hydrogen atoms were refined anisotropically, and all hydrogen atoms were placed in idealized positions and refined relatively isotropically with a riding model. Crystallographic data for **1** and **3** were deposited in the Cambridge Crystallographic Data Centre with the deposition numbers 1893702 and 1895292, respectively. Copies of the data can be obtained, free of charge, on application to the Director, CCDC, 12 Union Road, Cambridge CB21EZ, United Kingdom (fax: +44 (0)-1233-336033 or e-mail: deposit@ccdc.cam.ac.uk).

Crystal data for **1**: C_21_H_27_NO_5_, *M*_r_ = 373.44, monoclinic, *a* = 12.5780 (12) Å, *b* = 7.4893 (8) Å, *c* = 22.119 (2) Å, α = 90.00°, β = 95.281 (10)°, γ = 90.00°, *V* = 2074.7 (4) Å^3^, space group *C*2, *Z* = 4, *D*_x_ = 1.196 mg/m^3^, μ = 0.694 mm^−1^, and *F*(000) = 800. Crystal size: 0.08 mm × 0.07 mm × 0.07 mm. Reflections collected/unique: 6383/2896 [*R*(int) = 0.0556]. The final indices were *R*_1_ = 0.0607, *wR*_2_ = 0.1313 [*I* > 2σ(*I*)]. Flack parameter = 0.0 (5).

Crystal data for **3**: C_22_H_31_NO_5_, *M*_r_ = 389.22, monoclinic, *a* = 10.1548 (5) Å, *b* = 17.3448 (9) Å, *c* = 11.9203 (6) Å, α = 90.00°, β = 94.435 (4)°, γ = 90.00°, *V* = 2093.27 (19) Å^3^, space group *P*2_1_, *Z* = 4, *D*_x_ = 1.236 mg/m^3^, μ = 0.705 mm^−1^, and *F*(000) = 840. Crystal size: 0.12 mm × 0.11 mm × 0.11 mm. Reflections collected/unique: 8355/5693 [*R*(int) = 0.0199]. The final indices were *R*_1_ = 0.0459, *wR*_2_ = 0.1026 [*I* > 2σ(*I*)]. Flack parameter = 0.0 (2).

### Phytotoxicity Bioassays

The phytotoxicities of the isolated compounds against the seedling growth of amaranth (*Amaranthus retroflexus*) and lettuce (*Lactuca sativa*) were tested using a previously reported method (Zhang et al., 2013; Huang et al., 2018). Glyphosate was used as a positive control with concentrations of 200 and 50 μg/mL, respectively.

### Antibacterial and Antifungal Assays

The antibacterial and antifungal activities were evaluated using the conventional broth dilution assay (Appendino et al., 2008; Wang et al., 2016). Five phytopathogenic bacterial strains, including *Acidovorax avenae*, *Clavibacter michiganensis*, *Pseudomonas syringae*, *Ralstonia solanacearum*, and *Xanthomonas campestris*, and 12 phytopathogenic fungal strains, including *Alternaria alternata*, *Alternaria brassicicola*, *Aspergillus niger*, *Botrytis cinerea*, *Botryosphaeria dothidea*, *Colletotrichum* sp., *Fusarium graminearum*, *Fusarium oxysporum*, *Magnaporthe grisea*, *Pseudopestalotiopsis theae*, *Rhizoctonia cerealis*, and *Valsa mali* were used. Streptomycin sulfate, carbendazim, and prochloraz were used as positive controls for the bacteria and fungi, respectively.

TEM was performed to evaluate the effects of **5** (MIC and 4 × MIC) on *P. syringae* according to previously described methods (Liu et al., 2018; Zhao et al., 2018).

### Fermentation Optimization

The fungal strain *F. equiseti* D39 was cultured according to the OSMAC approach (Wang et al., 2017). To prepare the seed culture, a portion of fresh fungal mycelium was inoculated in potato dextrose water (PDW, 200 mL) culture medium with shaking (180 rpm) at 28°C for 3 days. For medium optimization, 500 mL baffled Erlenmeyer flasks, each containing a different medium (250 mL), were inoculated using the seed culture (5 mL) and grown at 28°C for 9 days with shaking (180 rpm). The seven media contained different crops, including carrots, maize kernels, malt, tuberous root of pachyrhizus, peanuts, potatoes, and soybean seeds, and were labeled as crops A–G. A portion of each product (200 g) was then boiled in water for 60 min, and the broth obtained was filtered through gauze. The infusion volume was subsequently adjusted to 1 L by adding distilled water, followed by the addition of glucose (20 g) to obtain the culture media. *F. equiseti* D39 was fermented in three 500-mL Erlenmeyer flasks each containing 250 mL crops A–G liquid medium and was shaken for 9 days at 28°C and 180 rpm. The culture media (750 mL) were then filtered to separate the broth from the mycelia, the broth samples were extracted three times with equal volumes of EtOAc. The mycelia were extracted twice with CH_2_Cl_2_–MeOH (v/v, 1/1), and then extracted three times with EtOAc after concentration. The organic extracts were combined and concentrated under vacuum, in order to get a total extract. The extracts were dissolved in MeCN to a final concentration of 10.0 mg/mL for HPLC analysis.

The production of **5** was estimated by establishing the standard curve between the HPLC peak areas and the concentrations of **5**. The standard curve was established using standard solutions of 0.05, 0.1, 0.2, 0.4, 0.6, and 1.0 mg/mL (sample size 10.0 μL) with a flow rate of 1.0 mL/min (MeCN/[H_2_O + 0.1% TFA], v/v, 60/40) on a C_18_ (Waters, 5 μm, 4.6 × 250 mm) column using a Waters e2695 separation module equipped with a Waters 2998 photodiode array detector. The linear curve and its fitting equation were established, and the production of **5** was calculated according to the fitting equation.

The optimal culture medium for production of **5** selected among crops A–G was further optimized with reference to salt concentration. In this respect, cultures were prepared in this medium after adjusting salinity at the levels of 1, 3, 5, 7, and 9% by adding natural sea salt. The procedure for determining the optimal fermentation salinity was as that described above.

## Results and Discussion

### Chemical Identification of the 3-Decalinoyltetramic Acids

**FIGURE 2:**
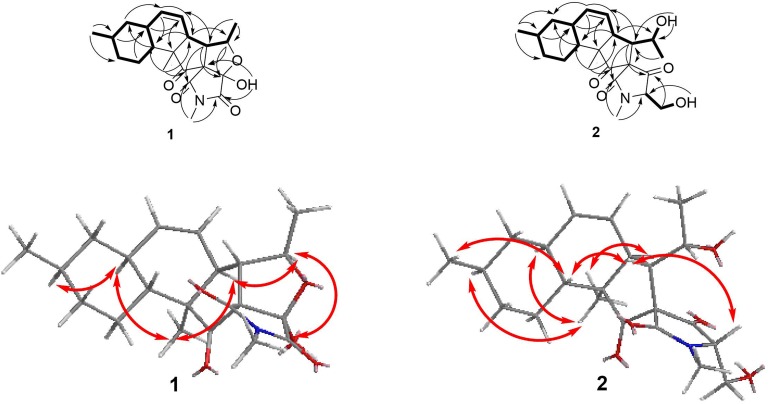
COSY, key HMBC, and NOESY correlations of **1** and **2**.

Fusarisetin C (**1**) was obtained as colorless crystals and assigned the molecular formula C_21_H_27_NO_5_ by HRESIMS, indicating nine degrees of unsaturation (Supplementary Figure S13). The ^1^H NMR spectrum in CD_3_OD (Table 1) displayed signals corresponding to two mutually coupled olefinic protons at δ_H_ 5.84 (ddd, *J* = 10.0, 4.5, 2.5 Hz) and 5.59 (d, *J* = 10.0 Hz), one oxymethine at δ_H_ 4.74 (dq, *J* = 6.5, 2.0 Hz), one *N*-methyl proton at δ_H_ 3.02 (s), two doublet methyl protons at 1.15 (d, *J* = 6.5 Hz) and 0.94 (d, *J* = 6.5 Hz), together with one singlet methyl proton at δ_H_ 0.98. Additionally, one exchangeable proton signal was observed in DMSO-*d*_6_ at δ 8.17 (s), and assigned as a hydroxyl proton (Table 1). The ^13^C NMR (Table 1) and DEPT spectra in DMSO showed resonances corresponding to three carbonyl groups including one ketone (δ_C_ 209.0) and two acylamides (δ_C_ 172.9, 172.6), eight methine carbons (δ_C_ 131.8, 124.5, 82.8, 56.8, 48.3, 37.4, 35.9, and 32.2), three quaternary carbons (δ_C_ 105.5, 73.3, and 53.2), three methylene carbons (δ_C_ 41.3, 34.8, and 24.7), one *N*-methyl carbon (δ_C_ 25.0), and three methyl carbons (δ_C_ 22.6, 22.2, and 13.8). These spectroscopic features suggested that **1** is a type of fusarisetin, possessing a carbon skeleton with a pentacyclic ring system comprising a decalin moiety (6/6) and a tricyclic moiety (5/5/5) (Supplementary Figures S3–S7, S9–S11). This structure is similar to fusarisetin B, which was isolated from the soil fungus, *Fusarium* sp. FN080326 (Jang et al., 2012). Compared to fusarisetin B, the disappearance of signals corresponding to oxymethylene and methine moieties, in addition to the appearance of an acylamide peak indicated that the -CHCH_2_OH group in the ring E of fusarisetin B was replaced by an acylamide carbonyl group in fusarisetin C. Indeed, the HMBC correlations from H-18 to C-2 and C-3, and from 4-OH to C-1 and C-3 confirmed the above conclusion (Figure 2). Detailed analysis of the HMQC, COSY, and HMBC spectra allowed the assignment of all carbon and proton resonances of **1**, and the planar structure of **1** was elucidated. In the NOESY spectrum, the correlations of H-21 with H-7 and H-10, and of H-10 with H-12, as well as of H-5 with H-7 and 4-OH indicated that these groups are positioned on the same face (Figure 2). These NOESY data and the relevant coupling constants confirmed the *trans* junction of the decalin ring system and the *cis* junction between the tricyclic and decalin ring systems(Supplementary Figures S8, S12). Thus, the relative configuration of **1** was thereby established as 1*R*′,4*R*′,5*R*′,6*S*′,7*S*′,10*S*′,12*R*′,15*R*′,16*S*′ since the 1*S*,4*R*′,5*R*′,6*S*′,7*S*′,10*S*′,12*R*′,15*R*′,16*S*′ combination does not lead to a reasonable model according to 3D simulations.

**FIGURE 3:**
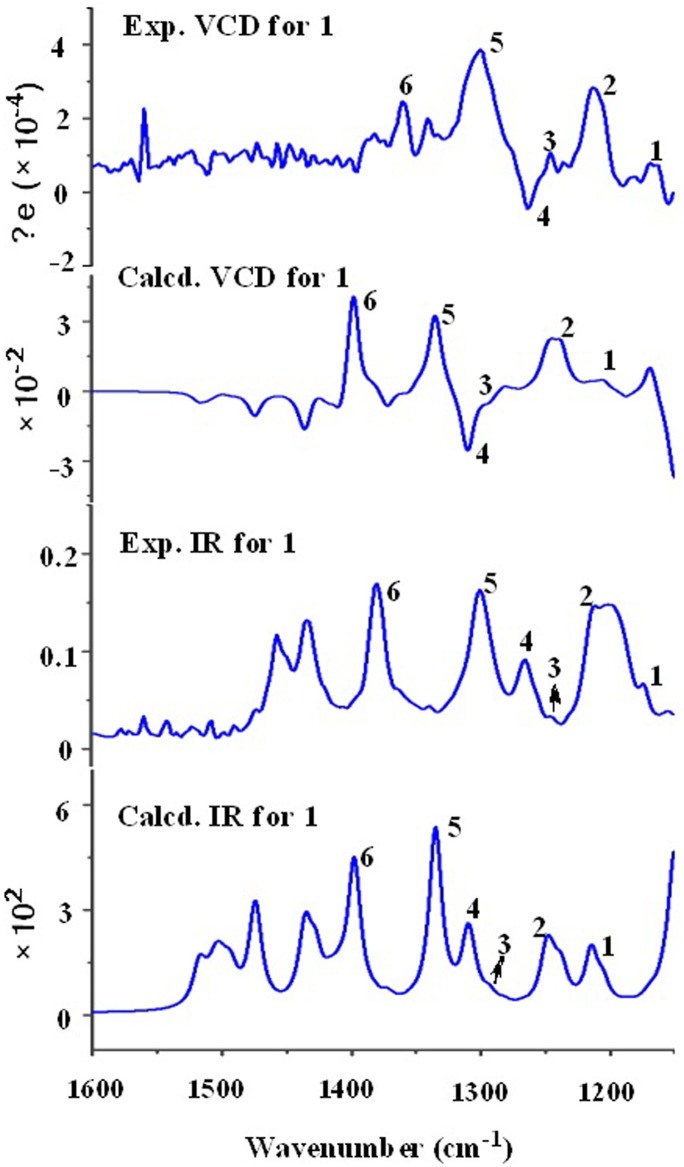
Comparison of the calculated VCD/IR spectra of (1*R*,4*R*,5*R*,6*S*,7*S*,10*S*,12*R*,15*R*,16*S*)-**1** and the experimental VCD/IR spectra of **1**.

**FIGURE 4:**
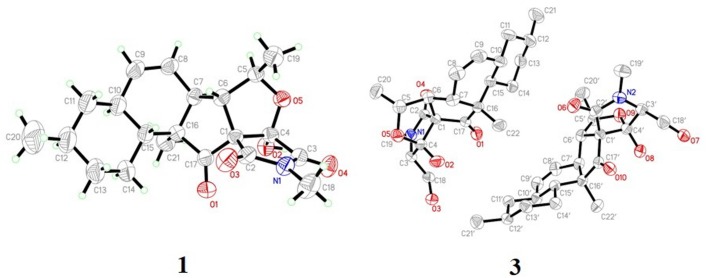
X-ray structure of **1** and **3**.

Recently, the VCD approach has become a robust and reliable alternative for the stereochemical characterizations of natural products (Sadlej et al., 2010; Keiderling and Lakhani, 2018). Thus, the experimental IR and VCD spectra of **1** (5.0 mg) were measured in DMSO-*d*_6_ (120 μL) using a BioTools dual PEM Chiral*IR*-2X spectrophotometer. The IR and VCD frequencies of (1*R*,4*R*,5*R*,6*S*,7*S*,10*S*,12*R*,15*R*,16*S*)**-1** were calculated at the MPW1PW91/6-311+G(d)//B3LYP/ 6-311+G(d) level of theory in the gas phase and the spectra were compared with the experimental IR and VCD spectra of **1** (Supplementary Table S1). As shown in Figure 3, all calculated IR and VCD signals of (1*R*,4*R*,5*R*,6*S*,7*S*,10*S*,12*R*,15*R*,16*S*)**-1** agreed with the experimental IR and VCD signals of **1**, thereby confirming the (1*R*,4*R*,5*R*,6*S*,7*S*,10*S*,12*R*,15*R*,16*S*) configuration for **1**. Fortunately, **1** was recrystallized from a mixture of dichloromethane/methanol (2:1) to yield crystals that were suitable for single-crystal X-ray diffraction. This confirmed the unambiguous assignment of the absolute configuration as 1*R*,4*R*,5*R*,6*S*,7*S*,10*S*,12*R*,15*R*,16*S* (Figure 4).

Fusarisetin D (**2**) was isolated as colorless oil possessing the molecular formula C_22_H_31_NO_5_ with eight degrees of unsaturation (Supplementary Figure S25). The general features of its ^1^H and ^13^C NMR spectra (Table 2) suggested that **2** is also a 3-decalinoyltetramic acid, and belongs to the family of fusarisetins. Analysis of the 1D and 2D NMR spectra indicated that the main differences between **2** and fusarisetin B could be found in the C, D, and E rings (Supplementary Figures S14–S18, S20–S22). The additional keto carbonyl signal and the disappearance of the C-4 oxymethine signal indicated that ring D was not present in **2**. In addition, the COSY cross-peaks of H-7/H-6/H-5/H-20, together with the HMBC correlations from H-6 to C-1, C-2, C-4, and C-8, and from H-20 to C-6 (Figure 2) reavealed that there was a –CH(OH)CH_3_ group anchored to C-6. The HMBC correlations from H-3 and H-18 to C-4 suggested that the C-O bond at C-4 in fusarisetin B was fractured in **2**. Thus, the planar structure of **2** was confirmed. Indeed, compound **2** represents the first 3-decalinoyltetramic acid, possessing an unprecedented carbon skeleton with a tetracyclic ring system comprising a decalin moiety (6/6) and a bicyclic moiety (5/5).

**FIGURE 5:**
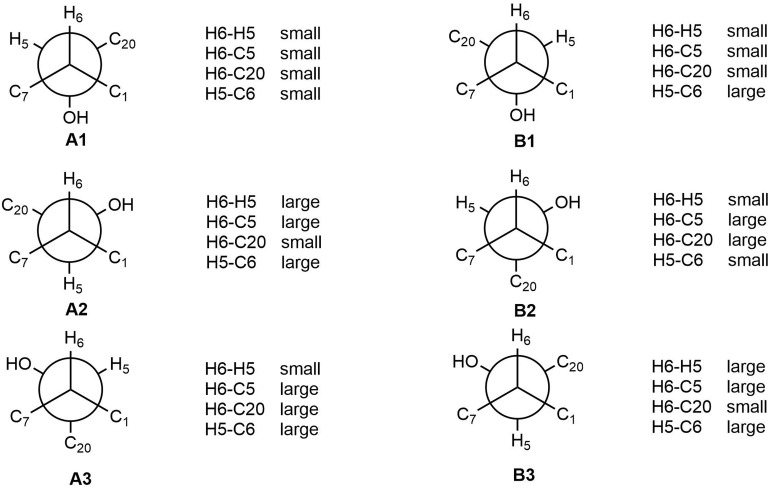
*J*-Based analysis of six hypothetical rotamers with 5*R*,6*S* **(A1–A3)** and 5*S*,6*S* **(B1–B3)** configuration to determine the stereochemistry of **2**.

The relative configuration of **2** (with the exception of C-1 and C-5) was then deduced on the basis of NOESY spectroscopic data (Figure 2) and Supplementary Figures S19, S23). The correlations of H-22 with H-7, H-10, and H-12 indicated that these groups are positioned on the same face. The correlations of H-6 with H-3 and H-15, and of H-15 with H-21 reaveled a *cis*-relationship. Furthermore, the relative configuration between C-5 and C-6 was determined by *J*-based configuration analysis (including ^2^*J*_C,H_ and ^3^*J*_C,H_ carbon-proton spin coupling constants, and proton-proton spin coupling constants ^3^*J*_H,H_) (Matsumori et al., 1999; Halecker et al., 2014) (Supplementary Figure S24). In acyclic systems such as the side chain of **2**, the configuration of adjacent asymmetric centers can be represented by staggered rotamers (Figure 5). Among the various configurations, four conformers, namely A-1, A-3, B-1, and B-2, could be identified using the ^3^*J*_H,H_, ^2^*J*_C,H_, and ^3^*J*_C,H_ values, while rotamers A-2 and B-3 could not be distinguished. The small coupling constants of ^3^*J* (H_5_,H_6_) = 1.5 Hz, ^2^*J* (H_5_, C_6_) = 1.8 Hz, and the large coupling constant of ^3^*J* (H_6_, C_5_) = 6.0 Hz, indicated an *anti*-like configuration between the proton and hydroxyl functions corresponding to B2 in Figure 4, thereby confirming the relative configuration of **2**.

Attempts were then made to determine the absolute configuration of **2** at the C-5 position using the modified Mosher’s method (Kusumi et al., 1991); however, neither the reactants nor the corresponding acylation products were detected, possibly because **2** was not stable under the alkaline conditions required for this technique. As the various natural fusarisetins included in the present study (i.e., **1**, **3–4**) have the same configuration in the A, B, and C rings, based on the biogenetic consideration, the absolute configuration of **2** should be 1*R*,3*S*,5*S*,6*S*,7*S*,10*S*,12*R*,15*R*,16*S*.

Known compounds **3–6** were identified as fusarisetin B (Ahn et al., 2012), fusarisetin A (Jang et al., 2012), equisetin (Singh et al., 1998), and *epi*-equisetin (Singh et al., 1998), by comparison of their spectroscopic data with the literature. Compound **3** was previously reported as its enantiomer in a patent, but was cited in another article as fusarisetin B, which corresponds with the structure determined herein (Ahn et al., 2012; Caro-Diaz et al., 2014). Although the structure of **4** was revised in 2012, that of **3** was not, and the determination of its absolute configuration was also not discussed. Fortunately, **3** was crystallized herein by evaporation from a solution of 20:1 MeOH/H_2_O over the course of 1 w, and the absolute configuration was determined to be 1*R*,3*S*,4*R*,5*R*,6*S*,7*S*,10*S*,12*R*,15*R*,16*S* (Figure 4).

### Plausible Biosynthetic Pathway Toward Compounds 1–4

**FIGURE 6:**
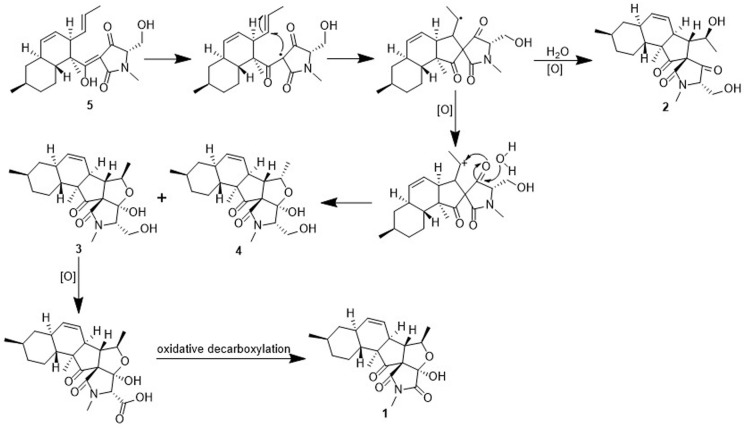
Proposed biosynthetic pathway of compounds **1**-**4**.

To the best of our knowledge, only two fusarisetins have been previously isolated from nature. Inspection of the fusarisetin and equisetin frameworks revealed that fusarisetins A–D may derive biogenetically from the oxidation of equisetin upon exposure to reactive oxygen species (ROS) (Figure 6; Xu et al., 2012; Yin et al., 2012). This biosynthetic scenario could account for the formation of a stabilized radical that, upon cyclization at the pendant alkene followed by trapping by ROS, could produce fusarisetins through single-electron oxidation and hemiketalization. Thus, the absolute configurations of the chiral centers in rings A, B, and C of fusarisetins A and D should be the same as those of fusarisetins B and C, which were confirmed by their X-ray structures (Figure 4).

**Table 3:** Anti-phytopathogenic bacterial and fungal activities of **5** and **6**.

Compunds	MIC (μM)				
	*C. michiganensis*	*P. syringae*	*A. brassicicola*	*F. graminearum*	*R. cerealis*
5	4.2	1.1	8.4	133.9	8.4
6	4.2	4.2	16.7	133.9	–
Streptomycin sulfate*^a^*	0.9	3.4	No test	No test	No test
Carbendazim*^b^*	No test	No test	–	8.2	16.3
Prochloraz*^b^*	No test	No test	0.4	No test	No test

*^a^Streptomycin sulfate was used as a positive control for antibacterial assays. ^b^Carbendazim and prochloraz were used as positive controls for antifungal assays. “–” means no antifungal activity.*

### Bioactivities of Compounds 1–6

3-Decalinoyltetramic acids and their derivatives have been found to exhibit a broad range of biological activities, including antiviral, antimicrobial, cytotoxic, and phytotoxic activities (Schobert and Schlenk, 2008; Mo et al., 2014). In the present study, all isolated compounds were subjected to a panel of bioassays to evaluate their potential activities. These included evaluation of their anti-phytopathogenic bacterial activities against *A. avenae, C. michiganensis*, *P. syringae*, *R. solanacearum*, and *X. campestris*, anti-phytopathogenic fungal activities against *A. alternata*, *A. brassicicola*, *A. niger*, *B. cinerea*, *B. dothidea*, *Colletotrichum* sp., *F. graminearum*, *F. oxysporum*, *M. grisea*, *P. theae*, *R. cerealis*, and *V. mali*, and phytotoxicities toward the seedling growth of amaranth (*A. retroflexus*) and lettuce (*L. sativa*). The corresponding results are outlined in Tables 3–5. As indicated, compounds **1–6** displayed obvious phytotoxicities, while compounds **5** and **6** also exhibited potent anti-phytopathogenic bacterial and fungal activities. Notably, compounds **5** and **6** showed remarkable antimicrobial activities against *P. syringae* and *R. cerealis*, with minimum inhibitory concentration (MIC) values of 1.1 and 8.4 μM, respectively, compared to 3.4 μM for streptomycin sulfate, and 16.3 μM for carbendazim. Interestingly, the equisetins have been widely reported for their antibacterial activities against Gram-positive bacteria (Schobert and Schlenk, 2008; Mo et al., 2014), and in this study, their remakable anti-Gram negative bacterial activity was also found. Moreover, compounds **4–6** exhibited a prominent phytotoxicity against growth of amaranth and lettuce seedlings at 200 μg/mL, and this strong phytotoxicity was still evident at lower concentrations (50 μg/mL). In addition, the fusarisetins were quoted as exhibiting acinar morphogenesis inhibitory activities (Ahn et al., 2012; Jang et al., 2012), and were reported to be phytotoxic for the first time in the present paper.

**Table 4:** Phytotoxicity of compounds **1–6** (200 μg/mL) toward seedling growth of amaranth and lettuce.

Strains	Root length (mm)		Hypocotyl length (mm)	

	Amaranth	Lettuce	Amaranth	Lettuce
1	4.60 ± 0.00	–	–	–
2	13.03 ± 0.32	–	–	–
3	7.65 ± 2.90	–	–	–
4	0	–	0	–
5	0	0	0	6.36 ± 0.59
6	0	0	0	4.90 ± 1.43
Glyphosate	0	0	0	3.75 ± 0.25
H_2_O	16.43 ± 1.55	20.94 ± 2.15	7.40 ± 0.77	8.40 ± 0.59

*The concentration of glyphosate was 200 μg/mL. A length <2.0 mm was regarded as no germination. “–” means no obvious effect on seedling growth.*

**Table 5:** Phytotoxicity of compounds **4–6** (50 μg/mL) toward seedling growth of amaranth.

Strains	Amaranth	
	Root length (mm)	Hypocotyl length (mm)
4	6.77 ± 1.93	6.30 ± 1.01
5	0	4.47 ± 1.29
6	0	5.20 ± 0.87
Glyphosate	0	0
H_2_O	16.43 ± 1.55	7.40 ± 0.77

*The concentration of glyphosate was 50 μg/mL. A length <2.0 mm was regarded as no germination.*

The effect of **5** on the cell membrane of *P. syringae* was then examined by TEM. The untreated cells appeared to be intact with the typical cellular organization (Supplementary Figure S1), while cells treated with **5** (1 × MIC and 4 × MIC) disintegrated, with this effect being more pronounced at higher concentration (Supplementary Figure S1). Indeed, compound **5** completely lysed the cell walls of the majority of cells, which were misshapen and disproportionate (Supplementary Figure S1). Hence, **5** treatment was confirmed to damage the cell membrane of *P. syringae* resulting in leakage of the intracellular components.

### Fermentation Optimization of 5

Due to the prominent bioactivities exhibited by compound **5**, the fermentation optimization was performed based on the OSMAC approach to improve its yield. Strain D39 was initially cultivated by SSF on rice medium, as it was reported that SSF is an efficient fermentation process in terms of producing complex metabolites due to its longer metabolic circle. In addition, SSF offers potential benefits for microbial cultivation for bioprocesses and product development (Dong et al., 2016). However, many fatty acids and other components from the solid culture medium could be mixed with those from the fungus during the extraction process, which can significantly affect the bioassays and chemical fingerprint assays. The long fermentation time of SSF is also a disadvantage, and so submerged flask fermentation (SmF) using seven different crop media was applied for the purpose of this study.

The standard curve of **5** was established by means of HPLC-UV measurements. Thus, the linear regression equation for **5** was determined to be y = 8.34 × 10^6^× -3.54 × 10^4^ (*R*^2^ = 0.99) (Supplementary Figure S2) (*R*^2^ = 0.99), where x is the concentration of **5** (mg/mL), and y is the peak area. All curves showed good linear relationships that could be used to estimate the production of **5** from the corresponding HPLC peak areas. The crude extracts were then subjected to HPLC analysis to determine the yields of **5**, which varied significantly (Supplementary Table S2). The results showed that the highest production of **5** was obtained using the crop F medium, yielding 21.61 mg/L.

Thus, the productivity of **5** was further optimized in the crop F medium by investigating the effects of salinity (i.e., 0, 1, 3, 5, 7, and 9%). It was found that the highest production of **5** was obtained under 1% salinity with a yield of 59.85 mg/L (Supplementary Table S2), and the production decreased upon increasing the salinity. Thus, crop F medium with a salinity of 1% was found to be the most favorable condition for the production of **5** from *F. equiseti* D39.

## Conclusion

In conclusion, we herein reported the isolation and identification of two novel fusarisetins (**1** and **2**), along with four known antimicrobial and phytotoxic analogs (**3–6**) from the marine-derived fungus *F. equiseti* D39. The absolute configuration of **1**, which was difficult to be determined by common means, such as ECD calculations and chemical conversions, due to the lack of chromophores and low yields, was determined by VCD method and verified by X-ray diffraction, suggesting a new horizon to define the absolute configurations of fusarisetins. The phytotoxicity of fusarisetins was reported for the first time in the present study. Furthermore, as the need for subsequent field trials, the OSMAC fermentation optimization approach toward the most bioactive compound **5** was employed, and the potato dextrose liquid medium with a salinity of 1% was found to be the most favorable, with a high yield of 59.85 mg/L. Due to the neglect on the study of agricultural biological activities of marine-derived fungi worldwide, the present study revealed that searching for new biopesticides from secondary metabolites of marine-derived fungi has a very broad prospect.

## Data Availability

The raw data supporting the conclusions of this manuscript will be made available by the authors, without undue reservation, to any qualified researcher.

## Author Contributions

DZ and CZ conceived and designed the experiments. XH, DW, ML, and JG performed the experiments. FC performed the VCD calculations. YP, JL, and YL analyzed the data. DZ wrote the manuscript. All authors reviewed the manuscript.

## Conflict of Interest Statement

XH, ML, JG, YP, and JL were employed by Zunyi Branch, Guizhou Tobacco Company. The remaining authors declare that the research was conducted in the absence of any commercial or financial relationships that could be construed as a potential conflict of interest.
