# Supplementary material for: Bioactive 3-Decalinoyltetramic Acids Derivatives From a Marine-Derived Strain of the Fungus Fusarium equiseti D39
Source: Front Microbiol. 2019 Jun 7;10:1285. doi: 10.3389/fmicb.2019.01285 (PMC6568041; doi:10.3389/fmicb.2019.01285)
Supplement: Supplementary file 1 [file Data_Sheet_1.ZIP › Supporting_Information/Supporting_Information .doc]

***Supporting Information***

# Bioactive 3-Decalinoyltetramic Acids Derivatives From a Marine-derivied Strain of the Fungus [*Fusarium equiseti*](http://www.baidu.com/link?url=UXpNy21Qq0L_mJ8YdGFwfHN0jQ6d-LMIN9kuzQuzq2yg0N0BkROqLTwCZgryWHxVTgiK4TebkbDogi6SEFO9XW3Qvf079DLUVbZusospxr_DEeeSgeBXeA3gFlM0vRABzabvw2DSavLl_ia6Yz2I1XUoLTX655JX1qfp4Rj_izqRVKRTKHAxqhnOj7bmTDkyzXvZOMO2WNIo3oFG2gemVeqbwRI62STj7MBpvnsmQ6LXH8XMI-o0ecYySc6HF84bENBbnfVspjYk6l9JsEJxKPNyzCzo4thiDsMTxdFOUdClBDuNURcif-t6BHi5K0w5E5YiZXma1uZ2J6H2GWQ3LuyMuofEH-TVZs2QjK61B2RwWYwo2CaX60yqz6u9t0NpgeacXYOMXqfZg13HJHA7hjnGKfpB1kh-DIPR6gWN1iy) D39

Donglin Zhao,^1†^ Xiaobin Han,^2†^ Dan Wang,^1^ Minghong Liu,^2^ Jianyu Gou,^2^ Yulong Peng,^2^ Jing Liu,^2^ Yiqiang Li,^1^ Fei Cao,^3^* and Chengsheng Zhang^1^*

^1^Marine Agriculture Research Center, Tobacco Research Institute of Chinese Academy of Agricultural Sciences, Qingdao 266101, People’s Republic of China

^2^Zunyi Branch, Guizhou Tobacco Company, Zunyi 563000, People’s Republic of China

^3^College of Pharmaceutical Sciences, Hebei University, Baoding 071002, People’s Republic of China

***Correspondence:**
Chengsheng Zhang
zhchengsheng@126.com

Fei Cao
zhchengsheng@126.com

^†^These authors contributed equally to this work.

**List of Supporting Information**

**Table S1.** The coordinate for the lowest-energy conformer [(1*R*,4*R*,5*R*,6*S*,7*S*,10*S*,12*R*,15*R*,16*S*)**-1**] in IR and VCD calculations

**Table S2.** The productions of **5** in different media

**Figure S1.** TEM images of *Pseudomonas syringae* cells treated with and without **5**

**Figure S2.** Standard curve of **5**

**Figure S3.** ^1^H NMR (500 MHz, CD_3_OD) spectrum of compound **1**

**Figure S4.** ^13^C NMR (125 MHz, CD_3_OD) spectrum of compound **1**

**Figure S5.** HMQC (CD_3_OD) spectrum of compound **1**

**Figure S6.** COSY (CD_3_OD) spectrum of compound **1**

**Figure S7.** HMBC (CD_3_OD) spectrum of compound **1**

**Figure S8.** NOESY (CD_3_OD) spectrum of compound **1**

**Figure S9.** ^1^H NMR (500 MHz, DMSO-*d*_6_) spectrum of compound **1**

**Figure S10.** ^13^C NMR (125 MHz, DMSO-*d*_6_) spectrum of compound **1**

**Figure S11.** HMBC (DMSO-*d*_6_) spectrum of compound **1**

**Figure S12.** NOESY (DMSO-*d*_6_) spectrum of compound **1**

**Figure S13.** HRESIMS spectrum of compound **1**

**Figure S14.** ^1^H NMR (500 MHz, CD_3_OD) spectrum of compound **2**

**Figure S15.** ^13^C NMR (125 MHz, CD_3_OD) spectrum of compound **2**

**Figure S16.** HMQC (CD_3_OD) spectrum of compound **2**

**Figure S17.** COSY (CD_3_OD) spectrum of compound **2**

**Figure S18.** HMBC (CD_3_OD) spectrum of compound **2**

**Figure S19.** NOESY (CD_3_OD) spectrum of compound **2**

**Figure S20.** ^1^H NMR (500 MHz, DMSO-*d*_6_) spectrum of compound **2**

**Figure S21.** ^13^C NMR (125 MHz, DMSO-*d*_6_) spectrum of compound **2**

**Figure S22.** HMBC (DMSO-*d*_6_) spectrum of compound **2**

**Figure S23.** NOESY (DMSO-*d*_6_) spectrum of compound **2**

**Figure S24.** HETLOC (DMSO-*d*_6_) spectrum of compound **2**

**Figure S25.** HRESIMS spectrum of compound **2**

**Table S1.** The coordinate for the lowest-energy conformer [(1*R*,4*R*,5*R*,6*S*,7*S*,10*S*,12*R*,15*R*,16*S*)**-1**] in IR and VCD calculations

----------------------------------------------------------------------------------

Coordinates (Angstroms)

X	Y	Z

-----------------------------------------------------------------------------------

C	4.73017400	-0.91530100	-0.27341000

C	3.61409000	-1.91113900	0.04218400

C	2.55455300	-1.32608900	0.97234800

C	1.97040200	-0.04643800	0.37714700

C	3.08987500	0.97774600	0.15248200

C	4.14104700	0.39413100	-0.79747500

C	0.79001200	0.58685300	1.17325600

C	0.22767900	1.77560800	0.36551600

C	1.28061700	2.64577300	-0.24641400

C	2.56099900	2.29262900	-0.33229900

C	-0.35979400	-0.41040300	1.16635900

C	-1.32821500	-0.05714400	0.02437800

C	-0.75650200	1.17103200	-0.67139700

C	-2.72531100	0.37417800	0.47828600

O	-3.12839000	1.30765800	-0.48533600

C	-1.99203200	2.03600100	-0.99302800

C	-1.62019500	-1.27670700	-0.83304500

N	-2.90345900	-1.71959900	-0.54915500

C	-3.59930400	-0.86610800	0.27965400

C	5.74831300	-1.49665300	-1.24457300

H	3.59732000	1.15130300	1.11470600

H	-0.36938100	2.38595000	1.05567100

H	1.58044900	-0.30461400	-0.61819100

C	1.16340500	0.96030000	2.60285500

O	-0.51524500	-1.33882400	1.90938800

H	-0.22140900	0.89514600	-1.58203900

C	-2.22173600	2.30344200	-2.46166500

O	-2.76766800	0.85926800	1.76906500

C	-3.45177500	-2.94139900	-1.09514500

O	-0.87081400	-1.80966000	-1.60914300

O	-4.69114800	-1.04236900	0.75154000

H	5.24853600	-0.68811700	0.66983200

H	4.04023000	-2.81734400	0.48550800

H	3.13738900	-2.22498700	-0.89582600

H	1.77053600	-2.06381800	1.15537600

H	3.00698400	-1.10959300	1.94702900

H	3.68296300	0.22352800	-1.78087800

H	4.94141700	1.12679200	-0.95442700

H	0.96253700	3.61445100	-0.62554200

H	3.27893000	2.98357700	-0.76966500

H	-1.93754000	2.98465000	-0.44650500

H	6.56151400	-0.79300000	-1.44310600

H	6.19367800	-2.41523800	-0.85317000

H	5.28165200	-1.74070000	-2.20401600

H	1.96965300	1.69602100	2.61930600

H	0.30456600	1.39360800	3.12000200

H	1.47757900	0.08437700	3.17047400

H	-1.40917800	2.91056000	-2.86771200

H	-3.15870200	2.84196900	-2.61263400

H	-2.26708600	1.36867400	-3.02491800

H	-3.68481200	0.84282500	2.06839500

H	-3.60914000	-2.84173000	-2.16968600

H	-2.76450000	-3.76641400	-0.91372800

H	-4.40166200	-3.13114000	-0.60035700

-----------------------------------------------------------------------------------

**Table S2. The productions of 5 in different media**

| Medium | Production (mg/L) |
| --- | --- |
| Crop A | 0.73 |
| Crop B | 1.15 |
| Crop C | 2.09 |
| Crop D | 10.27 |
| Crop E | 5.21 |
| Crop F | 21.61 |
| Crop G | 0.93 |
| Crop F 1% salinity | 59.85 |
| Crop F 3% salinity | 21.70 |
| Crop F 5% salinity | 9.32 |
| Crop F 7% salinity | 7.02 |
| Crop F 9% salinity | 2.14 |

**Figure S1.** TEM images of *Pseudomonas syringae* cells treated with **5** (a) 1 × MIC, (b) 4 × MIC, and (c) without **5**

**Figure S2.** Standard curve of **5**

**Figure S3.** ^1^H NMR (500 MHz, CD_3_OD) spectrum of compound **1**

**Figure S4.** ^13^C NMR (125 MHz, CD_3_OD) spectrum of compound **1**

**Figure S5.** HMQC (CD_3_OD) spectrum of compound **1**

**Figure S6.** COSY (CD_3_OD) spectrum of compound **1**

**Figure S7.** HMBC (CD_3_OD) spectrum of compound **1**

**Figure S8.** NOESY (CD_3_OD) spectrum of compound **1**

**Figure S9.** ^1^H NMR (500 MHz, DMSO-*d*_6_) spectrum of compound **1**

**Figure S10.** ^13^C NMR (125 MHz, DMSO-*d*_6_) spectrum of compound **1**

**Figure S11.** HMBC (DMSO-*d*_6_) spectrum of compound **1**

**Figure S12.** NOESY (DMSO-*d*_6_) spectrum of compound **1**

**Figure S13.** HRESIMS spectrum of compound **1**

**Figure S14.** ^1^H NMR (500 MHz, CD_3_OD) spectrum of compound **2**

**Figure S15.** ^13^C NMR (125 MHz, CD_3_OD) spectrum of compound **2**

**Figure S16.** HMQC (CD_3_OD) spectrum of compound **2**

**Figure S17.** COSY (CD_3_OD) spectrum of compound **2**

**Figure S18.** HMBC (CD_3_OD) spectrum of compound **2**

**Figure S19.** NOESY (CD_3_OD) spectrum of compound **2**

**Figure S20.** ^1^H NMR (500 MHz, DMSO-*d*_6_) spectrum of compound **2**

**Figure S21.** ^13^C NMR (125 MHz, DMSO-*d*_6_) spectrum of compound **2**

**Figure S22.** HMBC (DMSO-*d*_6_) spectrum of compound **2**

**Figure S23.** NOESY (DMSO-*d*_6_) spectrum of compound **2**

**Figure S24.** HETLOC (DMSO-*d*_6_) spectrum of compound **2**

**Figure S25.** HRESIMS spectrum of compound **2**
